# Biogeographic patterns and evolutionary history of *Elatostema* (Urticaceae)

**DOI:** 10.1186/s40529-025-00456-0

**Published:** 2025-03-19

**Authors:** Yu-Hsin Tseng, Alex K. Monro, Jer-Ming Hu

**Affiliations:** 1 Department of Life Sciences, National Chung Hsing University, 145 Xingda Rd., South Dist., Taichung, 40227 Taiwan; 2 Herbarium, Royal Botanic Gardens, Kew, London, TW9 3AE UK; 3 Institute of Ecology and Evolutionary Biology, National Taiwan University, Roosevelt Rd. Sec. 4 No. 1, Taipei City, 10617 Taiwan

**Keywords:** Ancestral area reconstructions, Boreotropics, Divergence time, Gene flow, Paleotropics, Tropical intercontinental disjunction

## Abstract

**Background:**

The paleotropics, home to half of Earth's rainforests, exhibit remarkable biodiversity and complex biogeographic patterns. Understanding the intercontinental distribution of plant taxa between Africa and Asia in this region is crucial for resolving longstanding debates on plant evolution and dispersal mechanisms. This study investigates the genus *Elatostema*, a widely distributed taxon found in subtropical and tropical Africa, Asia, and Australasia, aiming to elucidate the factors shaping its modern tropical disjunctions and evolutionary history.

**Result:**

Using molecular dating and ancestral area reconstruction, we reconstructed the historical biogeographic pattern of *Elatostema*. Our results indicated that the genus originated in tropical Asia during the Eocene, likely associated with boreotropical floras. The evolutionary history of *Elatostema* involved multiple intercontinental dispersal events, including two independent colonizations of Africa from Asia. Diversification within the core *Elatostema* clade was primarily driven by events in Asia and Oceania, with key factors contributing to this diversification including reciprocal dispersal between Malesia and Australasia, eastward island hopping and karstification in China. Furthermore, a geographical phylogenetic structure was observed within the core *Elatostema* clade, possibly due to limited seed and pollen dispersal.

**Conclusions:**

The present study provides the first comprehensive insights into the biogeography and evolution of *Elatostema*. The presence of numerous narrowly distributed endemics, relatively few widespread species, and geographical structures within *Elatostema* suggest that limited gene flow may be a crucial factor in speciation and evolutionary processes, similar to other species-rich genera.

**Supplementary Information:**

The online version contains supplementary material available at 10.1186/s40529-025-00456-0.

## Background

The paleotropics, including tropical Africa, Asia, and Oceania, comprise roughly half of the world’s tropical rainforests (Morley 2000) and have been recognized as significant biodiversity hotspots (Myers et al. 2000). Understanding the historical biogeographic connections across the paleotropics is a central theme in global plant biogeography (Raven and Axelrod 1974), which provides ideal models for investigating the long-lasting debate between vicariance and dispersal (Rieppei 2002). Biogeographic studies have revealed that multiple dispersal routes have significantly influenced the current distribution of paleotropical plants, particularly the intercontinental African-Asian distribution and species diversification in tropical Asia (Yuan et al. 2005).

Disjunct distribution of plant lineages in the paleotropics, a key challenge in plant biogeography (Raven and Axelrod 1974), is evident in numerous African-Asian plant genera (van Welzen et al. 2014; Zhou et al. 2012). Four primary hypotheses have been proposed to explain this phenomenon (Thomas et al. 2015). The first posits a Gondwanan vicariance origin, arising from the breakup of the Gondwana supercontinent. This scenario suggests that ancestral lineages became separated as India drifted northwards in the late Cretaceous (90–85 Ma), eventually colliding with Asian plate ca. 35 Ma (Ali and Aitchison 2008). Alternatively, the boreotropical migration hypothesis proposes that a widespread boreotropical flora during a warm phase peaking in the late Paleocene to early Eocene facilitated the expansion and dispersal of vegetation in Eurasia and Northern America, ca. 52–50 Ma (Wolfe 1975). This biotic exchange was likely driven by the expansion of forests in northern mid-latitudes (Wolfe 1975). Several studies have suggested that the African-Asian disjunctions may be attributed to dispersal events via Arabia during the Middle Miocene Climatic Optimum, ca. 17–15 Ma (Zachos et al. 2001). The formation of a land bridge between Africa and Southwest Asia provided opportunities for numerous organisms, including plant lineages (Thomas et al. 2015; Zhou et al. 2012) and primates (Bernor et al. 1987), to cross between Africa and Asia. Finally, the long-distance dispersal is also an important explanation for African-Asian disjunction and tropical forest biomes, especially in the case of recent speciation events, even though the dispersal mechanism remains largely unexplored (Zhou et al. 2012).

The Asian paleotropics harbor rich endemic species, shaped by complex geological and climatic history (Morley 2012; Woodruff 2010). This region has undergone significant Cenozoic transformations, including continental drift, plate collisions, mountain building, volcanic activity, and fluctuating landmasses (Wong 2011). Wong (2011) proposed two major dispersal scenarios and directions in this region: (1) a westward route across Wallace’s line from Australia and New Guinea into Sundaland, facilitated by organisms with winged seeds and tolerance of harsh conditions, potentially utilizing cool mountain corridors for migration (Morley 2000); and (2) an eastward migration from the Asian Mainland through Taiwan and the Philippines to Wallacea and New Guinea, eventually extension to Australia, New Zealand and Samoa, facilitated by island hopping hypothesis through a chain of volcanic islands (Wong 2011). Furthermore, three key periods—45, 25 and 5 Ma—marked by significant shifts in plate boundaries and movements caused by multiple collision events in Southeast Asia and Southwest Pacific, likely played a crucial role in shaping the formation and distribution of biota in this region (Hall 2002). Investigating the biogeographic and diversification processes of paleotropic biota within a robust phylogenetic framework will provide valuable insights into the assembly of the Asian paleotropical flora (Low et al. 2021).

*Elatostema* J.R. Forst. and G. Forst. (Urticaceae) is a taxonomically challenging group in Urticaceae, comprising over 650 species (POWO 2024) and thus representing a “big genus” defined by Frodin (2004). This diverse group includes understory herbs, subshrubs, and shrubs predominantly found in tropical and subtropical Asia and Africa, with a centre of species diversity in Southern China (Wang 2012). Because of homoplasy among diagnostic morphological characters, the inter- and infrageneric classifications of *Elatostema* have been controversial over a century, with differing delimitation for *Elatostema*, *Elatostematoides*, *Pellionia* and *Procris* (Schröter and Winkler 1935, 1936; Tseng et al. 2019; Wang 1980). Our recent phylogenetic analysis revealed three well-supported and morphologically distinct genera within *Elatostema* (*s.l.*): *Elatostema*, *Elatostematoides*, and *Procris* (Tseng et al. 2019).

Furthermore, our molecular phylogeny analyses indicate four major clades within *Elatostema*: core *Elatostema*, *Pellionia*, *Weddellia*, and African-*Elatostema*. The core *Elatostema* clade, comprising most *Elatostema* species, shows multiple diversification bursts across Asia and Oceania, with a notable hotspot in China and Southeast Asia. The *Pellionia* clade is mainly found in tropical and subtropical Asia and the Pacific. The *Weddellia* clade, characterized by opposite nanophyll, suggests that opposite leaves may be a plesiomorphy in *Elatostema s.l.* The African-*Elatostema* clade includes most African *Elatostema* species, but shares numerous morphological overlaps with the core *Elatostema* clade, making their reliable distinction challenging. Notably, *E. welwitschia* from Africa within core *Elatostema* clade is only one species falling outside the African-*Elatostema* (Tseng et al. 2019).

*Elatostema* exhibits an Asian-African disjunct distribution, with a wide geographic range, extending from Africa and Madagascar to Southern India, the tropical Himalayas, Indochina and Southern China, Philippines, Malaysia, and across Papuasia to Australia, Fiji, New Zealand and Polynesia. In addition, the genus also occurs in subtropical and sub-temperate areas of Taiwan, Korea and Japan. Wu et al. (2018) estimated this genus to have originated approximately 42 Ma. However, this estimation is based on a dataset including only ca. 30 *Elatostema* species, so the calibration time derived from this limited taxon sampling requires further validation. In addition, their study mainly focused on species from China and lacked any sampling from Africa and Southeastern Asia, leaving the mechanisms underlying the African-Asian disjunction and Asian paleotropics of *Elatostema* largely unresolved.

This study aims to elucidate the historical biogeography and investigate the directionality of intercontinental biotic exchange in *Elatostema*. To achieve this, we utilize a time-calibrated molecular phylogenetic framework integrated with ancestral area reconstruction. This approach will enable us to evaluate competing hypotheses regarding the origins of African-Asian disjunction and Asian paleotropical distribution of the genus. Specifically, this study will: (1) determine the timing and routes of key dispersal events, (2) identify the geological and climatic factors that have shaped the genus's evolutionary history, and (3) explore the potential mechanism driving diversification within *Elatostema*.

## Methods

### Taxon sampling

A total of 146 taxa were included in this study. Among these, 99 taxa of *Elatostema* were selected to represent the four major infrageneric clades: *Weddellia* (four taxa), *Pellionia* (eight taxa), African-*Elatostema* (six taxa) and core *Elatostema* (81 taxa) (clade nomenclature following Tseng et al. 2019). These samples exhibited a broad geographic distribution, spanning Africa, Madagascar, Asia and Oceania Island, and displayed various morphological forms. In addition, several other *Elatostema s.l.* taxa were chosen, including six species of *Elatostematoides*, five species of *Procris* and *Pellionia repens.* The representative species from four major clades (Clade I, Clade II, Clade III and Clade IV, clade nomenclature following Wu et al. 2013) of Urticaceae and seven species from Moraceae were included as outgroups (see Additional file 1 for detailed information).

### Calibration points selection

Although no *Elatostema* fossil has been recorded, some fossil records were found in Urticaceae. Among these, fossil fruits offer the most comprehensive fossil data in Urticaceae (Takhtajan 1982), so the calibration points of Urticaceae were determined to refer to the stratigraphic distribution of fruits. Considering the DNA sequences’ availability and fossils’ reliability, *Pilea* was chosen as the calibration point for the molecular dating analysis. Fruit fossils of *Pilea* have been documented from the former USSR (Takhtajan 1982), Bulgaria (Palamarev 1968; 1973; Palamarev and Petkova 1987), West Germany (Kirchheimer 1957) and Poland (Łańcucka-Środoniowa 1979). Most of these fossils were from the Miocene, with sporadic occurrence in the Eocene and Oligocene, and the oldest fossil of *Pilea* occurred in the late Eocene (ca. 39 Ma). Based on these fossil records, the crown node of *Pilea* was defined with a median of 39 and a 95% probability interval between 23 and 55 Ma, between the early Eocene to the late Oligocene (Collinson 1989).

Divergence time estimations can be biased from unbalanced taxon sampling, as Milne (2009) highlights. This study integrated the three secondary calibration points of Urticaceae taken from Wu et al. (2018; 2022) to address this potential issue. These points included: the divergent time of Urticaceae (68.7 Ma with a normal distribution to cover the 95% HPD range between 56.2 Ma and 87.1 Ma), the most recent common ancestor (MRCA) of Clade I and Clade IV (a mean of 54.9 Ma with a normal distribution to cover the 95% HPD range 53.9 Ma and 55.9 Ma), and Clade II + Clade III (a mean of 59.2 with a normal distribution to cover the 95% HPD range 58.2 Ma and 60.2 Ma).

### Molecular dating

This study employed a multi-locus approach, utilizing one nuclear (nrITS) and two chloroplast DNA sequences (*psbA-trnH* and *psbM-trnD*). Sequence data were mainly generated from our previous study and some sequences downloaded from GenBank. The detailed sequence information used in this study is provided in supplementary S1. Each marker was aligned using MAFFT v7.450 (Katoh and Standley 2013), followed by manually adjusted and assembled in Mesquite v.3.70 (Maddison and Maddison 2015).

Modeltest v.2.7 (Posada and Crandall 1998) was applied to determine the best model for nucleotide substitutions. The generalized time-reversible model (GTR + I + G model) was identified as the best-fit model. The divergence time estimation was conducted using two methods, the Bayesian method (BEAST v.2.7.5) (Bouckaert et al. 2019) and Penalized Likelihood (treePL) (Smith and O'Meara 2012). For BEAST analysis, input files were generated using BEAUti v.2.7.7 (Bouckaert et al. 2019). A Yule speciation model was selected as a tree prior, with an uncorrelated lognormal distributed (UCLD) relaxed clock model. The four calibration points were set based on the description in the above section. Two independent Markov chain Monte Carlo (MCMC) runs were conducted in the analyses, each with 1 ✕ 10^8^ generations, and the trees were sampled every 2000th generation. Time series plots of all parameters were analyzed in Tracer v.1.7.2 (Rambaut et al. 2018) to check for effective sample size values larger than 200. Trees were combined in LogCombiner v.2.7.5 (Bouckaert et al. 2019), setting the burn-in to 20% of the initial samples of each MCMC run. Finally, consensus trees with mean age estimates were calculated with TreeAnnotator v.2.7.5 (Bouckaert et al. 2019) and visualized with Figtree v.1.4.4 (Rambaut and Drummond 2010).

The treePL analysis followed the optimization guideline described by Maurin (2020). The maximum likelihood (ML) tree was reconstructed using the “ML + Through bootstrap” workflow of RAxML-HPC2 on XSEDE via the CIPRES Science Gateway. The ML tree was used as a constraint to generate 1000 rapid bootstrap replicates with branch lengths through the “Bootstrap + Consensus” workflow using the GTR + G model. Priming and cross-validation analyses were performed based on the best-ML tree. The best optimization parameters were implemented as follows: opt = 1, optad = 1, optcvad = 5. The cross-validation was iterated four times, ranging from 10–12 to 1012, to ascertain the best smoothing value, determined to be 10^–9^. Confidence intervals for the dated tree were obtained by conducting the treePL analysis with the bootstrap replicates using the same calibration points for BEAST setting, optimization, and cross-validation values as outlined above. The set of trees was summarized in TreeAnnotator v.2.7.5 (Bouckaert et al. 2019) with mean node heights and discarding the initial 10% as burn-in.

### Geographical inference

The geographic distribution for each species in the phylogeny of this study was determined based on published floras (Chen et al. 2003; Friis 1989; Hooker 1890; Reinecke 1898; Smith 1981; Tateishi 1993; Yang et al. 1996), taxonomic revisions (Tseng and Hu 2015; Wang 2014; Yahara 1984; Yang et al. 1995), local studies (Beaman 2001; Robinson 1910) and distribution data from the Global Biodiversity Information Facility (http://data.gbif.org/) and Plants of the World Online (https://powo.science.kew.org/) (POWO 2024). When the distribution information from these sources was unavailable, herbarium records or field collection conducted by authors were considered representative of the current distribution. The distribution of *Elatostema* was categorized into six distinct biogeographical areas following Takhtajan’s (1986) floristic regions: (A) continental Africa, (B) Madagascar; (C) India, including India, Bhutan, Sri Lanka and Nepal; (D) Indo-China; (E) East Asia, including China, Japan and Taiwan; (F) Malesia, including the Philippines, Indonesia, Malaysia, and Papua New Guinea; (G) Australasia, including Australia, New Zealand and Pacific Islands. Details on the species number and sampling extent within each biogeographical area are shown in Additional file 2.

Ancestral range and historical biogeographic events were reconstructed using the R package BioGeoBEARS implemented in RASP v.4.4 (Yu et al. 2020). Following the RASP manual guidelines (Yu et al. 2020), outgroups were excluded from the analysis and all species of *Elatostema*, *Elatostematodies* and *Procris* were included. One thousand randomly chosen post burn-in trees derived from the BEAST analysis were used and biogeographical results were summarized on the BEAST consensus tree. Geographic distributions of each species were coded using the above criteria. Our data were evaluated in DEC, DIALIKE, and BAYAREALIKE models with and without the founder event speciation parameter “j” using AIC to find the best-fit model and likelihood ratio tests were conducted to assess the statistical significance of model selection.

## Results

### Phylogenetic analysis

The aligned length of nrITS was 1402 bp (792 variable sites, 597 parsimony-informative characters), 799 bp of *psbA-trnH* (451 variable sites, 299 parsimony-informative characters) and 929 bp of *psbM-trnD* (441 variable sites, 278 parsimony-informative characters), respectively. The total length of the combined dataset was 3130 bp, containing 1,684 variable sites and 1174 parsimony-informative characters.

**Fig. 1:**
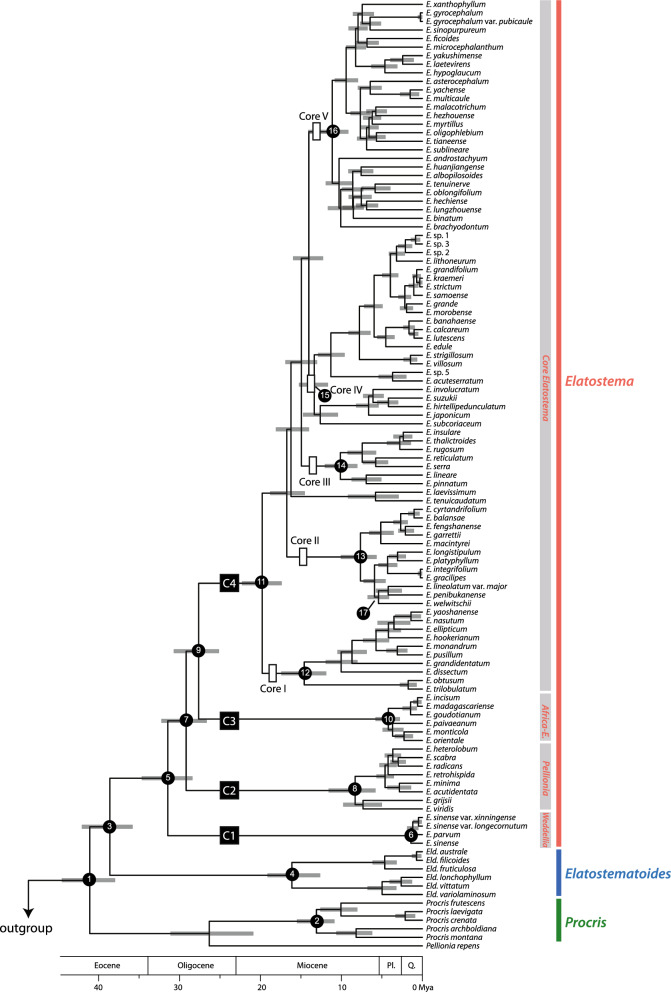

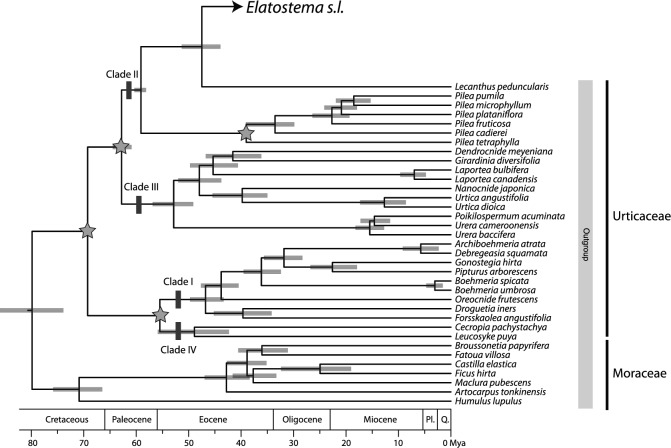
Chronogram of *Elatostema* and related taxa based on a combined data set of one nuclear (nrITS) and two chloroplast markers (*psbA-trnH*, *psbM-trnD*). The phylogeny was inferred using treePL, with scale bars representing 95% highest posterior density (HPD) interval and all posterior probabilities equal to one. The star signs denote calibration points employed for molecular dating. Clade names within *Elatostema* and Urticaceae follow the nomenclature proposed by Tseng et al. (2019) and Wu et al. (2018), respectively

**Fig. 2:**
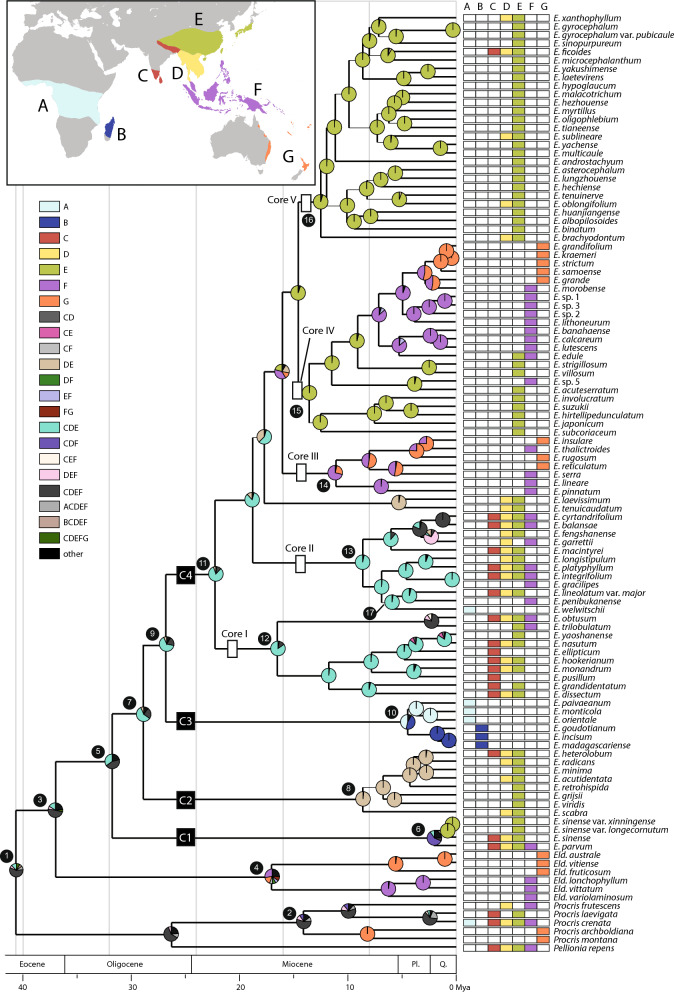
Ancestral area reconstructions inferred by BioGeoBEARs overlaid on the maximum clade credibility chronogram estimated with BEAST. Pie charts at internal nodes represent the relative probabilities of ancestral area reconstruction. Thin branches indicate nodes with posterior clade probabilities < 0.9. Colored squares adjacent to the terminal names correspond to their geographic distributions with the seven biogeographic regions defined in this study. The map shows these regions, each identified by a unique letter and color. Clade names within *Elatostema* follow the nomenclature proposed by Tseng et al. (2019)

Both BEAST and treePL analyses generated congruent backbone phylogenies for the Urticaceae family, recovering four major clades (Clade I, II, III, VI) as found in Wu et al. (2018). Similarly, for *Elatostema s.l.*, both BEAST and treePL also produced well-resolved results, largely consistent with the topologies from Maximum Parsimony (MP), Maximum Likelihood (ML) and Bayesian Inference (BI) trees presented in our previous study (Tseng et al. 2019), but with conflicts in some nodes, especially in tip nodes. These results strongly support that *Elatostema s.l.* can be divided into three well-supported genera (*Procris*, *Elatostematoides* and *Elatostema*) (PP = 1.0 for all three clades in both methods). Within *Elatostema* (Clade C, Figs. 1 and 2), four subclades (Clade C1, C2, C3, C4, named as *Weddellia*, African-*Elatostema*, *Pellionia*, core *Elatostema* clade, Figs. 1 and 2) showed strong support (PP = 1.0 for all four clades in two methods). Furthermore, the core *Elatostema* clade was divided into five major subclades designated as Core I, II, III, IV, and V (node 12—node 16) (Figs. 1 and 2).

### Divergence time estimation

Divergence time estimation using different age calibration methods showed similar results (Table 1), with discrepancies generally less than two million years and substantial overlap in their credible intervals. Consequently, for consistency, the dating results presented herein are based on treePL method, as shown in Fig. 1.

**Table 1:** Age estimates and ancestral area reconstructions for key nodes

Node number and Taxa	Age estimates				
	Beast (Yule model) Mean (Ma)	95% HPD (Ma)	TreePL Mean (Ma)	95% HPD (Ma)	Ancestral area (probability)
1. *Elatostema s.l* crown	40.65	34.78–46.38	41.09	38.04–44.47	CDEF (0.49)
2. *Procris* crown	14.11	8.55–20.12	13.09	10.93–15.42	CDEF (0.55)
3. *Elatostema* stem	37.0	31.30–42.72	38.61	35.86–42.0	CDEF (0.49)
4. *Elatostematoides* crown	17.04	9.38–25.09	16.12	12.69–19.07	F (0.28)
5. *Elatostema* crown	31.73	26.40–37.11	31.45	28.47–34.6	CDE (0.35), CDEF (0.44)
6. *Weddellia* crown	2.08	0.71–3.90	1.42	0.81–2.11	CDE (0.59)
7. *Pellionia* stem	28.88	23.64–33.88	29.19	26.7–32.17	CDE (0.59), CDEF (0.25)
8. *Pellionia* crown	8.60	5.10–12.69	8.3	5.85–11.51	DE (0.98)
9. African-Elatostema stem	26.79	23.64–33.88	27.64	25.19–30.65	CDE (0.65)
10. African-Elatostema crown	4.47	2.31–6.81	4.21	2.83–5.72	A (0.46), B (0.46)
11. Core Elatostema crown	22.29	18.25–26.50	19.79	17.44–22.22	CDE (0.82)
12. Core I crown	16.51	12.45–20.91	14.59	11.94–17.36	CDE (0.85)
13. Core II crown	8.61	5.69–12.03	7.65	5.71–10.0	CDE (0.97)
14. Core III crown	11.06	7.44–14.76	10.03	8.09–11.98	F (0.71), G (0.25)
15. Core IV crown	13.58	10.93–16.48	13.36	11.73–15.17	E (0.99)
16. Core V crown	11.98	9.26–14.72	11.13	9.21–14.04	E (0.95)
17. Asian-African dispersal	5.93	3.4–8.26	5.42	4.18–6.7	CDE (0.98)

Node numbers correspond to those labelled in Figs. 1 and 2. Letters represent the seven biogeographic regions defined in this study, with corresponding ancestral area reconstructions and their associated probabilities (> 0.2)

*Elatostema s.l.* began diversifying in the Eocene (41.09 Ma, node 1, Fig. 1 and Table 1). The estimated divergence time of *Procris* was similar to Wu et al. (2018) (13.09 vs. 13.6 Ma, node 2). This study presents the first calibration estimate for *Elatostematoides*, which originated in the Eocene (38.61 Ma, node 3) and diverged in the Miocene (17.04 Ma, node 4). The origin of *Elatostema* could be traced back to the early Eocene (38.61 Ma, node 3), with the initial divergence in the Oligocene (31.45 Ma, node 5). This was followed by the establishment of four clades (C1, C2, C3, C4) during the Oligocene to Pleistocene (31.45–1.42 Ma). The *Weddellia* clade (C1) was the earliest originated lineage within *Elatostema* (31.45 Ma, node 5, Fig. 1 and Table 1), with diverging around 1.42 Ma (node 6, Fig. 1). The *Pellionia* (C2) and African-*Elatostema* (C3) clades both originated in Oligocene (29.19 and 27.64 Ma, node 7 and node 9) and subsequently diverged from late Miocene and Pliocene (8.3 and 4.21 Ma, node 8 and node 10). The crown diversification in the core *Elatostema* clade (C4) began in the early Miocene (19.79 Ma, node 11), with at least five significant diversification bursts in Miocene (node 12–node 16) and one dispersal event from Asia to Africa (5.42 Ma, node 17).

### Geographic distribution

The results revealed a strong association between geographic distribution and phylogenetic relationship in African*-Elatostema* and the core *Elatostema* clades. African*-Elatostema* clade comprised two subclades (node 10, Fig. 2): one including three species from exclusively continental Africa and the other subclade including three endemic species restricted to Madagascar. In the core *Elatostema* clade, 23 out of 27 species in Core V (node 16) were distributed in East Asia (Area E). Meanwhile, taxa and Core II (node 13) and a few clades of Core III (node 14) were found in Malesia or Australasia (Area F or G).

BioGeoBEARS analysis found BAYAREALIKE + J as the best-fitting model for ancestral area reconstruction. The divergent time, node support and ancestral area reconstruction results at nodes of interest are shown in Table 1 and Fig. 2. Based on the results, *Elatostema* likely originated in Asia (Area CDE|CDEF, with a relative probability of 0.35| 0.44, node 5, Fig. 2). The diversification of *Weddellia* (Area CDF, node 6) and *Pellionia* clades (Area DE, node 8) also occurred in Asia.

Two intercontinental dispersal events from Asia to Africa paleotropics were identified: one occurred during the Oligocene to Pliocene (27.64–4.21 Ma, node 9–10, Fig. 2) and the other during the late Miocene (ca. 5 Ma, node 17). A potential dispersal event between Africa and Madagascar in African-*Elatostema* occurred during the late Oligocene to the late Miocene (ca. 4 Ma, node 10).

**Fig. 3:**
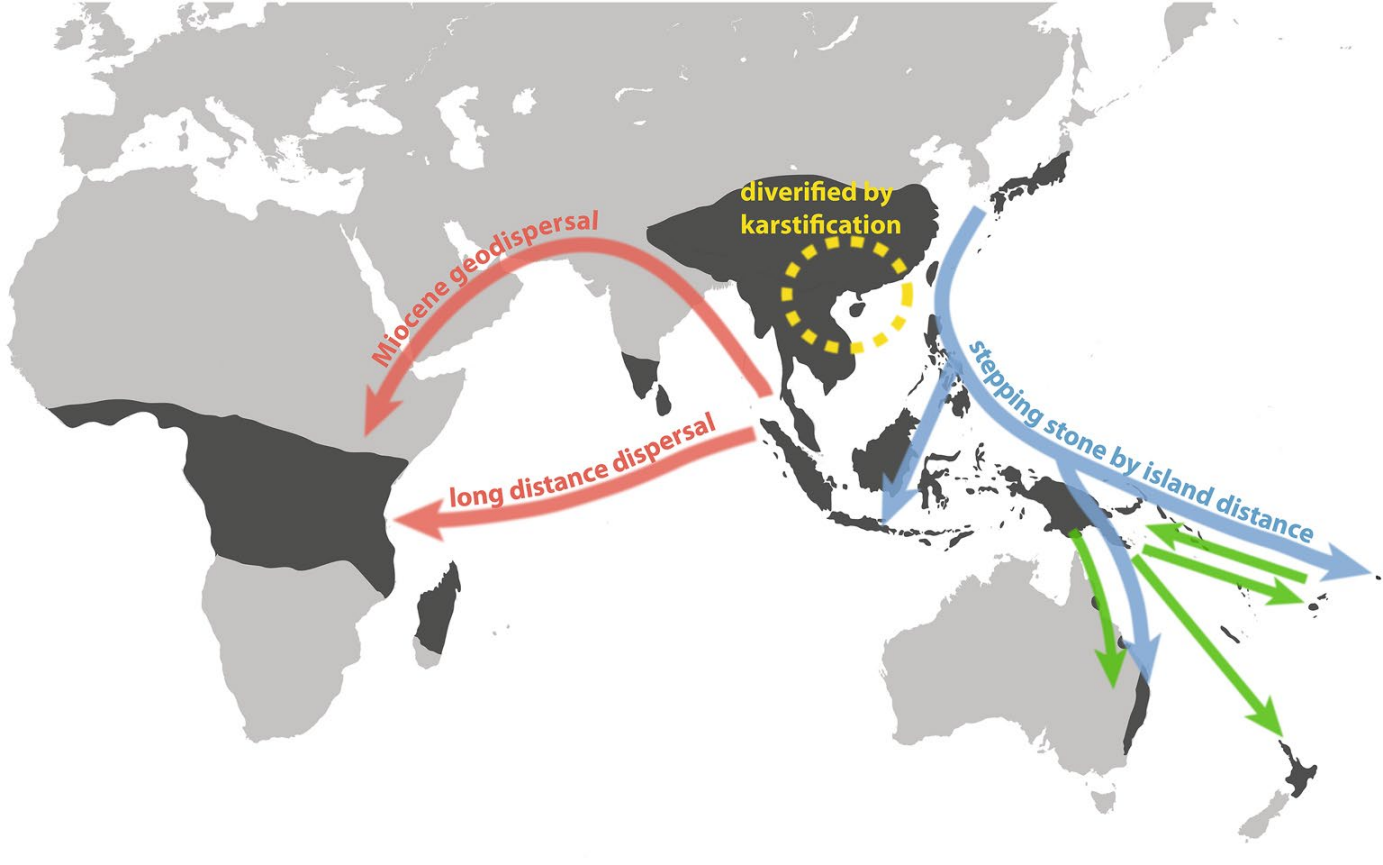
The biogeographic history of *Elatostema* is characterized by several significant dispersal events, including Miocene geodipersal and long-distance dispersals from Asia to Africa, diversification driven by karst landscapes in China, stepping-stone dispersal from East Asia through Malesia to Australasia, and reciprocal dispersal events between Malesia and Australasia

Five major diversification bursts (Core I, II, III, IV, V) identified in the core *Elatostema* clade were accompanied by numerous dispersal events. Core I and Core II diversified mainly in Asia (Area CDE), especially in India, Indo-China and East Asia, while species diversification in Core V was restricted to East Asia (Area E). Core III (node 14) likely originated in Malesia (Area F) with several back-and-forth dispersal events between Malesia to Australasia, extending to the regions of Australia (represented by *E. reticulatum*), Fiji (*E. insulare*) and New Zealand (*E. rugosum*), followed by a reverse dispersal event from Australasia back to Sabah and Sarawak (*E. thalictrolides*). In Core IV, the eastward directional dispersal event from East Asia occurred (node 15), potentially crossing through Lanyu, the Philippines, reaching Borneo and New Guinea, and further extending to Australia and Samoa. The major putative dispersal routes in *Elatostema* are summarized in Fig. 3.

## Discussion

### Origin and diversification of *Elatostema*

Our time-calibrated phylogeny suggests an Eocene origin (38.61 Ma) for *Elatostema* likely in tropical Asia, slightly more recent than the estimate of Wu et al. (2018) (ca. 42 Ma). The discrepancy probably results from differences in taxon sampling. Our study, comprising a broader sampling and employing both BEAST and treePL, provides greater confidence in our ages estimated and ancestral area reconstruction.

While largely congruent with the topology of Tseng et al. (2019), our phylogeny exhibits some topological incongruences. The African-*Elatostema* clade and *Pellionia* clade, identified as sister groups in Tseng et al. (2019), are not monophyletic here. Some conflicts are also observed at several tip nodes. Although our ingroup sampling remains largely consistent with that of Tseng et al. (2019), our study differs significantly in ourgroup selection. Tseng et al. (2019) included only six species to represent the major clades of Urticaceae (following Wu et al. 2013). In contrast, our study incorporated a more extensive outgroup sampling, comprising 28 species of Urticaceae and seven species of Moraceae. As highlighted by Grant (2019), outgroup selection can profoundly influence sequence alignment and phylogenetic tree reconstruction, affecting tree topology, ingroup rooting, and potential long-branch attraction. Moreover, Donoghue et al. (1989) emphasized that the effect of adding taxa depends on the strength of existing character evidence, with weakly supported clades being particularly susceptible to changes in taxon sampling. In Tseng et al. (2019), African-*Elatostema* and *Pellionia* clades were recovered as a monophyletic group with no to moderate support values. Given this weak support, the addition of a more comprehensive outgroup in our study may have altered their inferred relationships. A similar pattern of topological discordance is also observed at other tip nodes. It is important to note that the differing phylogenetic placements of African-*Elatostema* and *Pellionia* clades may directly impact biogeographic inference. Therefore, these incongruences necessitate further investigation using more expanded molecular data, such as plastome or Hyb-Seq.

The Eocene origin of *Elatostema* likely coincides with the formation of boreotropical flora, an extensive frost-free and humid climate belt during a warm phase of the late Paleocene to Eocene. Following its origin, African-*Elatostema Pellionia*, *Weddellia* clades experienced a long evolutionary stasis, characterized by a significant temporal gap (20.89–30.03 Ma) between their estimated stem and crown ages. This phenomenon also observed in other plants (e.g., *Cycas* and *Hedyosmum*) (Antonelli and Sanmartin 2011; Nagalingum et al. 2011), may be attributed to low diversification, extinction events, or climatic cooling. Subsequent diversification may have been triggered by climatic shifts, such as global climate change (Antonelli and Sanmartin 2011; Nagalingum et al. 2011). As observed in *Pellionia*, this clade diversified in the Miocene, potentially triggered by the global temperate optimum during the middle Miocene that occurred around 17–15 Ma (Zachos et al. 2001). Although the *Weddellia* and African-*Elatostema* clades did not exhibit a clear warm-induced diversification signal, their low speciation rates may reflect sustained cooling over time.

### African-Asian disjunct distribution in *Elatostema*: Dispersal via Arabia and long-distance ocean current

Our study revealed at least two independent dispersal events contributing to the African-Asian disjunct distribution in *Elatostema*: the first one correlated with the Asian origin of the African-*Elatostema* clade in the Oligocene and the second one within the core *Elatostema* clade (*E. welwitschii*) from tropical Asia to continental Africa in the late Miocene.

Four major hypotheses have been proposed to explain Asia–Africa disjunctions (Thomas et al. 2015). However, the Gondwana vicariance hypothesis, triggered by the breakup of Gondwana in the Middle Jurassic, India separating from Madagascar in the late Cretaceous (90–85 Ma) and followed by India’s northeastwards drift and collision with the Asian plate (50–35 Ma), occurs too early to account for the origin of late Oligoene and Miocene of these two events. Similarly, the boreotropical paleoflora (ca. 50 Ma) formation is correlated with the origin of *Elatostema* in Eocene, so this hypothesis also predates late Oligoene and Miocene African-Asian dispersal events in *Elatostema*.

The late Oligocene origin of the African-*Elatostema* clade coincides with the Late Oligocene Warming to the end of the Mid-Miocene Climatic Optimum, lending support to the Miocene geodisperal hypothesis. During this interval, tectonic activities facilitated the formation of a land bridge linking the Arabian Peninsula and the Anatolian plate (Röegl 1998), enabling *Elatostema* to potentially disperse from Asia to Africa via Arabia. The absence of *Elatostema* species in Arabia or North Africa to date is likely because of the extensive aridification in the late Miocene and the early Pliocene (Thomas et al. 2015).

The tiny seed and fruit size of *Elatostema,* with the absence of a documented animal dispersal vector and the lack of wind dispersal syndrome*,* suggests ocean currents as the potential mechanism for more recent long-distance dispersal, as found in dispersal event of *E. welwitschii*. This hypothesis is supported by Wu et al. (2018), who demonstrated the feasibility of transoceanic seed dispersal for numerous Urticaceae species, including *Elatostema stewardii*, highlighting adaptations for seawater survival. While this mechanism likely facilitated the colonization of *E. welwitschii* from Asia, the involvement of a single species contrasts with the diversification observed within the African-*Elatostema* clade. This difference may be attributed to the cooling trend at the end of the Miocene, potentially limiting diversification opportunities. Furthermore, a relatively shorter saltwater tolerance for *Elatostema* (only 108 days, Wu et al. 2018), compared to many species Urticaceae species (over 220 days or even ten months), may have restricted the frequency of successful long-distance dispersal events.

### Dispersal history of *Elatostema* in Malesia and Australasia: evidence for eastward island hopping and tectonic influence

Ancestral area reconstruction reveals at least two key dispersal events shaping the distribution of *Elatostema* in Malesia and Australasia. The first event, within Core III (node 14) during the Miocene, likely originated in Malesia, involving multiple back-and-forth dispersal events between Malesia and Australasia. The second route, within Core IV during the Miocene (node 15), involved an eastward dispersal from East Asia to Malesia and Australasia. The early divergent lineage of this route includes five species endemic to East Asia, suggesting an East Asian origin, likely Japan or Taiwan. This scenario supports an island hopping hypothesis proposed by Wong (2011), which posits a dispersal route originating in Japan or Taiwan, moving eastward through Lanyu, the Philippines, Borneo, New Guinea and further extending to Australia and Samoa.

Tectonic activity also played a crucial in facilitating these dispersal events. The northward shifts and strike-slip motions of Philippine Sea Plate approximately 10–15 Ma (Hall 2002), likely enabled the dispersal of *Elatostema* from East Asia to the Philippines during a concurrent time interval. Similarly, the late Miocene dispersal event from western Malesia (represented by Java and Borneo) to eastern Malesia (represented by New Guinea) coincides with the emergence of volcanic islands along the Sunda and Banda arcs (Hall 2001, 2009), suggesting that these volcanic formations likely acted as critical stepping stones for dispersal across eastern Malesia.

These findings highlight the significant roles of island hopping and tectonic activity as drivers of *Elatostema*’s diversification in Malesia and Australasia. Such processes likely created novel niches, promoting allopatric speciation within *Elatostema*.

### Diversification dynamics of *Elatostema* in Karst ecosystems

The Core V (node 16) with the core *Elatostema* clade represents a distinct diversification pattern, characterized by remarkable species proliferation, particularly in China. While inhabiting diverse habitats including damp, shady streamside and forest ground, *Elatostema* has demonstrated a remarkable ability to thrive in the challenging environment of limestone karst formations along the Sino-Vietnamese border. These species successfully colonize both the entrance and twilight zones of karst caves (Fu et al. 2017; Monro et al. 2018). This study focused on 23 of 55 documented *Elatostema* inhabiting limestone regions of China (Fu et al. 2020), mainly originating during the Miocene, a period of significant tectonic activity and intensified rock erosion, accelerating the karstification process (Liu 1997a, 1997b, 2003). This temporal congruence suggests a strong association between the dynamic evolution of the karst landscape and the diversification of these limestone-dwelling *Elatostema* species. Further investigations, including expended sampling and in-depth ecological studies, are necessary to elucidate the mechanisms driving the high diversity and endemism of *Elatostema* within these unique karst ecosystems.

### Limited correlation between morphology and biogeography

A strong correlation between biogeographic distribution and morphological variation appears unlikely within *Elatostema*. The African-*Elatostema* and core *Elatostema* clades exhibit significant overlap in key morphological characteristics, hindering the identification of reliable diagnostic traits to distinguish between them (Tseng et al. 2019). Moreover, substantial homoplasy is observed in morphological characters within the geographically widespread core *Elatostema* clade, rendering them unreliable for predicting species origins. However, certain morphological traits, such as shrubby growth forms, nanophylls, and specific inflorescence structures (e.g., absence of a receptacle and involucre in female flowers), are notably absent in African species. These characters are more commonly observed in the *Pellionia* and *Weddellia* clades. This observation suggests that both dispersal events from Asia to Africa likely originated from within the core *Elatostema* clade, potentially from lineages largely lacking these specific morphological features.

### Evolution in *Elatostema*

Our result reveals a strong geographical monophyly in *Elatostema*, likely driven by its limited dispersal ability. Seed dispersal in *Elatostema* relies on a unique mechanism involving inflexed staminodes, which typically restricts dispersal to short-distance (Corlett 2009). Field observations confirm this, with seed dispersal distance rarely exceeding one meter. Constrained by the absence of effective long-distance dispersal vectors, *Elatostema* populations often remain in small and isolated areas, contributing to the geographical monophyly. In addition, *Elatostema*, characterised by small flowers, appears to be wind-pollinated. However, unlike most wind-pollinated species adapted to open and arid habitats (Culley et al. 2002; Stephens et al. 2023), *Elatostema* typically inhabits shaded, sheltered and moist environments, which may limit pollen transport and further reinforcing the pattern of geographical monophyly in *Elatostema*.

Limited gene flow may be a potential driver of speciation, a pattern evident in *Begonia*, a genus of more than 2000 species with similarly limited dispersal (Hughes et al. 2015). According to Hughes and Hollingsworth (2008), this constraint underpins geographical monophyly, high levels of endemism, and the relatively scarce presence of widely distributed species in *Begonia*. Even its few widespread species exhibit significant population substructure due to limited gene flow (Hughes and Hollingsworth 2008; Twyford et al. 2013).

*Elatostema* also exhibits comparable trends. For example, in China, approximately 89.2% of *Elatostema* species are endemic (Wang 2014). Similarly, high levels of endemism occur in Sabah (75.0%) (Beaman 2001), Japan (50.0%) (Tateishi 1993), and Taiwan (33.3%) (Tseng and Hu 2015; Yang et al. 1995). Furthermore, the relatively widespread *Elatostema* species are often restricted to few clades and display marked morphological variation across their range, reflecting local ecological adaptations. For example, *E. obtusum*, a widespread species, shows regional variations in stem and hair morphology: some populations have hairs on the four-angled stems (*E. obtusum* var. *obtusum*), while others have hairs confined to the two furrowed edges of semi-terete stems (*E. obtusum* var. *trilobulatum*). Despite these morphological variations, these two varieties are closely related, highlighting a recent evolutionary divergence. This pattern of geographical monophyly, high endemism and morphological variation with widespread species parallels trends observed in *Begonia* (Hughes and Hollingsworth 2008). Consequently, limited gene flow may be key drivers of diversification of *Elatostema*, contributing to its emergence as a big genus (Frodin 2004).

*Pilea*, the largest genus in Urticaceae with over 700 species (Fu et al. 2022; Monro 2004), shares striking diversification patterns with *Elatostema*. *Pilea* also exhibits geographical monophyly (Fu et al. 2022; Monro 2006), high levels of endemism (38.8% in China and 38.5% in Taiwan) (Chen et al. 2003; Yang et al. 1996) and limited gene flow due to limited dispersal. These parallel phenomenon between *Elatostema* and *Pilea* suggests that similar evolutionary processes may drive their speciation and diversification. In contrast, their sister taxa, *Elatostematoides* (20 species) and *Lecanthus* (2 species), are relatively species-poor. Future comparative studies of dispersal ability and gene flow between the species-rich genera and species-poor sister genera could shed light on the mechanisms underlying this disparity, thereby enhancing our understanding of speciation in Urticaceae.

### Biogeographic study in Urticaceae genera

Fossil evidence suggests that diversification within Urticaceae peaked during the Eocene to the Miocene (Collinson 1989), a period characterized by significant climatic shifts—from the early Eocene thermal maximum to the Eocene–Oligocene colling and the middle Miocene Climatic Optimum (Zachos et al. 2001). This study confirms that the origin and diversification of *Elatostema* were closely linked to these Cenozoic climatic changes.

This study presents the first biogeographic analysis of *Elatostema*, providing insights into the potential causes of the African and Asian disjunction. Several other Urticaceae genera, including *Australina*, *Droguetia*, *Girardinia*, and *Urera*, also exhibit intercontinental disjunctions, but they are still unexplored. Applying the similar methods employed in this study to these other genera can significantly advance our understanding of the mechanisms underlying these disjunctions and the broader biogeographic patterns within Urticaceae.

## Conclusions

Plate tectonic movement and global climate change patterns significantly influence the dispersion patterns observed in numerous tropical flowering plant groups (Morley 2000). Through comprehensive analyses integrating divergent time assessments, ancestral area reconstructions, and biogeographic data, our study unveils a complex evolutionary history for *Elatostema*. This pioneering biogeographic investigation of *Elatostema* represents a significant step forward in unravelling the evolutionary trajectory of this genus. These findings enhance our understanding of *Elatostema*'s evolution and contribute substantially to the broader knowledge of plant biogeography in Urticaceae.

## Supplementary Information

Additional file 1.

Additional file 2.

## Data Availability

GenBank accession numbers of DNA sequences and species names analyzed in this study are shown in the supplementary information (Additional file 1). Species number and sampling in this study of each biogeographical area are shown in Additional file 2.

## Abbreviations

BI: Bayesian Inference
Ma: Million years ago
MP: Maximum Parsimony
ML: Maximum Likelihood
PP: Posterior probability
