# Supplementary material for: Biogeographic patterns and evolutionary history of Elatostema (Urticaceae)
Source: Bot Stud. 2025 Mar 19;66:10. doi: 10.1186/s40529-025-00456-0 (PMC11923331; doi:10.1186/s40529-025-00456-0)
Supplement: Supplementary file 1 — Additional file 1. [file 40529_2025_456_MOESM1_ESM.pdf]

Supplementary information

Additional file 1

Species names and GenBank accession numbers of DNA sequences used in this study. Voucher data is given using the following format: Taxon name, collection locality, GenBank accession numbers for ITS, *psbA-trnH*, *psbM-trnD*, respectively.

Elatostema s.l.

Elatostema

Elatostema acuteserratum B.L.Shih & Yuen P.Yang, KP858860, KP858673, KP858600. *Elatostema albopilosoides* Q.Lin & L.D.Duan, KP858885, KP858722, KP858577. *Elatostema androstachyum* W.T.Wang, A.K.Monro & Y.G.Wei, KP858871, KP858711, KP858575. *Elatostema asterocephalum* W.T.Wang, KP858878, KP858712, KP858533. *Elatostema balansae* Gagnep., China, KP858847, KP858696, KP858559. *Elatostema banahaense* C.B.Rob., Philippines, KP858815, KP858662, *KP858592. Elatostema binatum* W.T.Wang & Y.G.Wei, China, KP858895, KP858726, KP858518. *Elatostema brachyodontum* (Hand.-Mazz.) W.T.Wang, KP858888, KP858708, KP858557. *Elatostema brachyodontum* (Hand.-Mazz.) W.T.Wang, China, KP858889, KP858709, KP858573. *Elatostema calcareum* Merr., KP858817, KP858650, KP858589. *Elatostema cyrtandrifolium* (Zoll. & Moritzi) Miq., KP858844, KP858700, KP858558. *Elatostema cyrtandrifolium* (Zoll. & Moritzi) Miq., KP858845, KP858700, KP858558. *Elatostema dissectum* Wedd., KP858832, KP858740, KP858607. *Elatostema edule* C.B. Rob., KP858818, KP858664, KP858591. *Elatostema edule* C.B. Rob., KP858819, KP858663, KP858590. *Elatostema ellipticum* Wedd., NA, KP858774, KP858641. *Elatostema fengshanense* W.T.Wang & Y.G.Wei, KP858848, KP858694, KP858530. *Elatostema ficoides* Wedd., KP858870, KP858703, KP858522. *Elatostema garrettii* Yahara, KP858849, KP858698, KP858535. *Elatostema goudotianum* Wedd., KP858798, KP858749, KP858629. *Elatostema grande*

(Wedd.) P.S.Green, KP858821, KP858598, KP858598. *Elatostema grandidentatum* W.T.Wang, KP858833, KP858737, KP858608. *Elatostema grandifolium* Reinecke, KP858821, KP858659, KP858595. *Elatostema gyrocephalum* W.T.Wang & Y.G.Wei, KP858886, KP858727, KP858570. *Elatostema gyrocephalum* W.T.Wang & Y.G.Wei var. *pubicaule* W.T.Wang & Y.G.Wei, KP858887, KP858728, KP858578. *Elatostema hechiense* W.T.Wang & Y.G.Wei, China, KP858875, KP858729, KP858537. *Elatostema hezhouense* W.T.Wang, Y.G.Wei & A.K.Monro, KP858881, KP858705, KP858538. *Elatostema hirtellipedunculatum* B.L.Shih & Yuen P.Yang, KP858863, KP858671, KP858568. *Elatostema hookerianum* Wedd., KP858904, KP858735, KP858605. *Elatostema huanjiangense* W.T.Wang & Y.G.Wei, KP858875, KP858730, KP858537. *Elatostema hypoglaucum* B.L.Shih & Yuen P.Yang, KP858891, KP858718, KP858516. *Elatostema incisum* Wedd., KP858799, KP858750, KP858630. *Elatostema insulare* A.C.Sm., KP858839, KP858681, KP858554. *Elatostema integrifolium* (D.Don) Wedd., KP858853, KP858677, KP858553. *Elatostema involucratum* Franch. & Sav., KP858866, KP858672, KP858574. *Elatostema japonicum* Wedd., KP858867, KP858715, KP858566. *Elatostema kraemeri* Reinecke, KP858824, KP858657, KP858596. *Elatostema laetevirens* Makino, Japan, KP858893, KP858683, KP858524. *Elatostema laevissimum* W.T.Wang, KP858861, KP858687, KP858562. *Elatostema lineare* Stapf, KP858835, KP858713, KP858546. *Elatostema lineolatum* var. *majus* Wedd., KP858855, KP858689, KP858551. *Elatostema lithoneurum* Stapf, KP858827, KP858653, KP858588. *Elatostema longistipulum* Hand.-Mazz., KP858856, KP858679, KP858552. *Elatostema lungzhouense* W.T.Wang, KP858868, KP858716, KP858565. *Elatostema lutescens* C.B.Rob., KP858816, KP858651, KP858585. *Elatostema macintyrei* Dunn, KP858850, KP858695, KP858560. *Elatostema madagascariense* Wedd., KP858800, KP858752, KP858631. *Elatostema malacotrichum* W.T.Wang & Y.G.Wei, KP858876, KP858710, KP858544. *Elatostema microcephalanthum*

Hayata, KP858874, KP858706, KP858579. *Elatostema microcephalanthum* Hayata, KP858873, KP858707, KP858556. *Elatostema monandrum* (Buch.-Ham. ex D.Don) H.Hara, KP858905, KP858734, KP858606. *Elatostema monticola* Hook.f., KP858803, KP858690, KP858628. *Elatostema morobense* L.M.Perry, KP858820, KP858661, KP858593. *Elatostema multicaule* W.T.Wang, Y.G.Wei & A.K.Monro, KP858884, KP858685, KP858520. *Elatostema myrtillus* Hand.-Mazz., KP858880, KP858704, KP858523. *Elatostema nasutum* Hook.f., KP858902, KP858741, KP858603. *Elatostema oblongifolium* Fu, KP858897, KP858724, KP858545. *Elatostema oblongifolium* Fu, KP858898, KP858723, KP858526. *Elatostema obtusum* Wedd., KP858901, KP858739, KP858513. *Elatostema obtusum Wedd.* var. *trilobulatum* (Hayata) W.T.Wang, KP858900, KP858738, KP858515. *Elatostema oligophlebium* W.T.Wang, Y.G.Wei & L.F.Fu, KP858877, KP858721, KP858548. *Elatostema orientale* Engl., KP858802, KP858753, KP858627. *Elatostema paivaeanum* Wedd., KP858801, KP858751, KP858626. *Elatostema paivaeanum* Wedd., Malawi, *E. G. Chapman 103* (BM), KP858804, KP858754, KP858625. *Elatostema parvum* (Blume) Blume ex Miq., KP858794, KP858744, KP858620. *Elatostema penibukanense* Gibbs, KP858854, KP858693, KP858542. *Elatostema pinnatum* R.Beaman, KP858836, KP858719, KP858521. *Elatostema platyphyllum* Wedd., KP858858, KP858678, KP858540. *Elatostema platyphyllum* Wedd., KC420490, KC420500, KP858541. *Elatostema pusillum* C.B.Clarke ex Hook.f., KP858906, KP858736, KP858602. *Elatostema reticulatum*, KP858834, KP858714, KP858555. *Elatostema rugosum* Wedd., NA, KP858773, KP858640. *Elatostema samoense* Reinecke, KP858826, KP858660, KP858594. *Elatostema serra* H.J.P.Winkl., KP858837, KP858720, KP858534. *Elatostema sinense* H.Schroet., KP858797, KP858747, KP858619. *Elatostema sinense* H.Schroet. var. *longicornutum* (H.Schroet.) W.T.Wang, KP858795, KP858745, KP858622. *Elatostema sinense* H.Schroet. var. *xinningense* (W.T.Wang) L.D.Duan & Q.Lin, KP858796, KP858746, KP858621. *Elatostema*

sinopurpureum W.T.Wang, KP858869, KP858686, KP858532. *Elatostema* sp. 1, KP858829, KP858648, KP858586. *Elatostema* sp. 2, KP858830, KP858652, KP858580. *Elatostema* sp. 3, KP858831, KP858649, KP858587. *Elatostema* sp. 4, KP858828, KP858654, KP858581. *Elatostema* sp. 5, KP858859, KP858674, KP858601. *Elatostema* sp. 6, KP858851, KP858675, KP858547. *Elatostema strictum* Reinecke, KP858825, KP858658, KP858597. *Elatostema strigillosum* B.L.Shih & Yuen P.Yang, KP858813, KP858667, KP858583. *Elatostema strigillosum* B.L.Shih & Yuen P.Yang, KP858814, KP858669, KP858584. *Elatostema subcoriaceum* B.L.Shih & Yuen P.Yang, KP858894, KP858646, KP858539. *Elatostema sublineare* W.T.Wang, KP858882, KP858682, KP858514. *Elatostema suzukii* T.Yamaz., KP858864, KP858666, KP858525. *Elatostema suzukii* T.Yamaz., KP858865, KP858665, KP858576. *Elatostema tenuicaudatum* W.T.Wang, KP858862, KP858688, KP858528. *Elatostema tenuinerve* W.T.Wang & Y.G.Wei, KP858896, KP858717, KP858572. *Elatostema thalictroides* Stapf, KP858838, KP858680, KP858536. *Elatostema tianeense* W.T.Wang & Y.G.Wei, KP858879, KP858647, KP858569. *Elatostema villosum* B.L.Shih & Yuen P.Yang, KP858812, KP858668, KP858582. *Elatostema welwitschii* Engl., KP858840, KP858692, KP858543. *Elatostema welwitschii* Engl., KP858841, KP858691, KP858550. *Elatostema xanthophyllum* W.T.Wang, KP858890, KP858702, KP858571. *Elatostema yachense* W.T.Wang, Y.G.Wei & A.K.Monro, KP858883, KP858684, KP858519. *Elatostema yakushimense* Hatus., KP858892, KP858670, KP858517. *Elatostema yaoshanense* W.T.Wang, KP858903, KP858742, KP858604. *Pellionia acutidentata* W.T.Wang, KP858777, KP858759, KP858638. *Pellionia grijsii* Hance, KC420491, KC420504, KP858635. *Pellionia heteroloba* Wedd., KP858806, KP858756, KP858634. *Pellionia minima* Makino, KP858809, KP858757, KP858636. *Pellionia radicans* Wedd., KP858810, KP858758, KP858637. *Pellionia radicans* Wedd., KP858811, KP858755, KP858632. *Pellionia repens* (Lour.) Merr., KU161129, KU161131, KU161130. *Pellionia*

retrohispida W.T.Wang, KP858808, KP858760, KP858639. *Pellionia scabra* Benth., KC420492, KC420503, KP858624. *Pellionia viridis* C.H.Wright, KP858805, KP858748, KP858633.

Elatostematoides

Elatostematoides australe Hallier f., Fiji, KP858790, KP858732, KP858612. *Elatostematoides fruticulosa* K.Schum., KP858788, KP858733, KP858610. *Elatostematoides lonchophyllum* H.Schroet., KP858793, KP858761, KP858512. *Elatostematoides variolaminosum* H.Schroet. var. *latum* H.Schroet, KP858791, KP858762, NA. *Elatostematoides filicoides* (A.Gray ex Wedd.) A.C.Sm., KP858789, KP858731, KP858611. *Elatostematoides vittatum* Hallier f., KP858792, KP858763, KP858609.

Procris

Procris archboldiana A.C.Sm., KP858785, KP858769, KP858614. *Procris crenata* C.B.Rob., KP858782, KP858766, KP858616. *Procris crenata* C.B.Rob., KP858783, KP858767, KP858617. *Procris frutescens* Blume, KP858781, KP858764, KP858618. *Procris laevigata* Blume, KP858784, KP858765, KP858615. *Procris montana* (Endl.) Steud., NA, KP858768, KP858613.

Outgroup:

Moraceae

Artocarpus tonkinensis A.Chev. ex Gagnep., NA, NA, MZ379793. *Castilla elastica* Cerv., FJ916997, HG963693, NA. *Ficus hirta* Vahl, KX055723, OQ612627, OQ612627. *Humulus lupulus* L., MH712704, PP230458, PP230458. *Maclura pubescens* (Trécul) Z.K.Zhou & M.G.Gilbert, KP093095, KP095812, NA.

Urticaceae

Archiboehmeria atrata (Gagnep.) C.J.Chen, KF137798, NA, NA. *Boehmeria spicata* (Thunb.) Thunb., KF835857, NC_036989, NC_036989. *Boehmeria umbrosa* (Hand.-Mazz.) W.T.Wang, KF835858, MF990291, MF990291. *Broussonetia papyrifera* (L.) L'Hér. ex Vent., HM623778, NA, NA. *Cecropia pachystachya* Trécul, ON479724, NC_039763, NC_039763. *Debregeasia squamata* King ex Hook.f., NA, NA, MN189959. *Dendrocnide meyeniana* (Walp.) Chew, NA, NA, NC_064738. *Droguetia iners* (Forssk.) Schweinf., KM586476, MN189960, MN189960. *Fatoua villosa* (Thunb.) Nakai, KF137858, KC285025, NA. *Forsskaolea angustifolia* Retz., KF137861, NC_062309, NC_062309. *Girardinia diversifolia* (Link) Friis, NA, NA, NC_064969. *Gonostegia hirta* (Blume) Miq., NA, NA, NC_053931. *Laportea bulbifera* (Sieb. & Zucc.) Wedd., NA, NA, NC_063590. *Laportea canadensis* (L.) Wedd., NA, NA, NC_064747. *Lecanthus peduncularis* (Royle) Wedd., KP858787, NA, NA. *Leucosyke puya* (Hook.) den Baaker & Mabb., OM892763, NC_064751, NC_064751. *Nanocnide japonica* Blume, KP858907, KP 858645, KP858511. *Oreocnide frutescens* (Thunb.) Miq., NA, NA, NC_062322. *Pellionia repens* (Lour.) Merr., KU161129, KU161131, KU161130. *Pilea cadierei* Gagnep. & Guillaumin, MT516348, NC_054343, NC_054343. *Pilea fruticosa* Hook.f., DQ175604, KP858642, KP858505. *Pilea microphylla* (L.) Liebm., NA, NA, NC_054344. *Pilea plataniflora* C.H.Wright, NA, NA, NC_056134. *Pilea pumila* A.Gray, NA, NA, NC_054345. *Pilea tetraphylla* (Steud.) Blume, MZ490600, NA, NA. *Pipturus arborescens* C.B.Rob., KF137908, MN189967, MN189967. *Poikilospermum acuminatum* (Trécul) Merr., KP858908, KP858644, KP858510. *Urera baccifera* (L.) Gaudich. ex Wedd., OM892780, OM761935, OM761935. *Urera cameroonensis* Wedd., OM892781, OM877278, OM877278. *Urtica angustifolia* Fisch. ex Hornem., NA, NA, NC_064944. *Urtica dioica* L., KF971193, KF971259, NC_064948.
