# Supplementary material for: Biogeographic patterns and evolutionary history of Elatostema (Urticaceae)
Source: Bot Stud. 2025 Mar 19;66:10. doi: 10.1186/s40529-025-00456-0 (PMC11923331; doi:10.1186/s40529-025-00456-0)
Supplement: Supplementary file 2 — Additional file 2. [file 40529_2025_456_MOESM2_ESM.pdf]

Additional file 2

Species number and sampling in this study of each biogeographical area. Species number is based on the estimation of Wang (2014).

Locality	Defined area	Species number	Sampling
Continental Africa	A	10	4
Madagascar	B	4	3
India	C	50	13
Indo-China	D	50	14
East Asia	E	290	57
Malesia	F	220	25
Australasia	G	40	8
